# ﻿Redescription of *Parascorpaenamoultoni* (Whitley, 1961) (Actinopterygii, Scorpaenidae), with new distribution records for the species

**DOI:** 10.3897/zookeys.1219.134970

**Published:** 2024-12-04

**Authors:** Roxanne Cabebe-Barnuevo, Kunto Wibowo, Hiroyuki Motomura

**Affiliations:** 1 The United Graduate School of Agricultural Sciences, Kagoshima University, 1-21-24 Korimoto, Kagoshima 890-0065, Japan Kagoshima University Korimoto Japan; 2 Museum Zoologicum Bogoriense, Research Center for Biosystematics and Evolution, National Research and Innovation Agency, Cibinong 16911, Indonesia Museum Zoologicum Bogoriense Cibinong Indonesia; 3 The Kagoshima University Museum, 1-21-30 Korimoto, Kagoshima 890-0065, Japan The Kagoshima University Museum Korimoto Japan

**Keywords:** COI, morphology, new record, *Scorpaenamcadamsi*, *Scorpaenamoultoni*, scorpionfish

## Abstract

Although the status of *Parascorpaenamoultoni* (Whitley, 1961) is now well established, the morphology of the species has been re-examined, with new diagnostic features identified. Typically 15 or 16 pectoral-fin rays are present, together with two suborbital ridges, each with a single spine and the origin of the first ridge posterior to the second, well-developed interorbital ridges forming a loop, an undeveloped occipital pit, no scales on the dorsal- and anal-fin soft ray bases. The known range of the species includes Taiwan, the Philippines, Micronesia, Indonesia, Timor-Leste, Papua New Guinea, Solomon Islands, Vanuatu, and Fiji in addition to previously reported Australia, New Caledonia, and Japan.

## ﻿Introduction

*Scorpaenamoultoni* Whitley, 1961 was originally described from a single specimen collected north of Wilson Island, Capricorn Group, Queensland, Australia, in a depth of ca 15 m. Allen and Cross (1989) later reported the species from the Great Barrier Reef, Queensland. Although Allen et al. (2006) synonymized *S.moultoni* with *Parascorpaenamcadamsi* (Fowler, 1938), without significant justification, Motomura et al. (2011) later validated the taxonomic status of the former, reinstating it as a distinct species, and reported an additional record which extended the known distribution range to New Caledonia. Fricke et al. (2011) provided a detailed distribution within New Caledonia, encompassing Grand Passage (Îles Bélep and northern lagoon) and Grande Terre (northern and southern regions), and subsequent reports from Japan (Tsuno et al. 2022; Mochizuki et al. 2023) have further documented the species off various islands. Based on these published reports, *P.moultoni* has been considered as widely distributed in the western Pacific, from the Ryukyu Islands north to Kochi Prefecture in Japan, and southward to northern Australia and New Caledonia.

This study addresses discrepancies between the original description of *P.moultoni* by Whitley (1961) and features observed in examined specimens, so as to provide a revised morphological description for more accurately distinguishing *P.moultoni* from other valid species within the genus *Parascorpaena*. The examination of many voucher specimens confirmed the wide distribution of *P.moultoni* across the western Pacific Ocean (see below).

## ﻿Materials and methods

Counts and measurements followed Motomura (2004a, 2004b), Motomura et al. (2005a, 2005b, 2005c, 2006a, 2006b, 2009), and Motomura and Johnson (2006). Standard length (**SL**), head length (**HL**), and morphometrics were measured to the nearest 0.1 mm using digital calipers. Head spine terminology follows Motomura et al. (2009) and Wibowo and Motomura (2021). Curatorial procedures for KAUM specimens followed Motomura and Ishihara (2013).

Phylogenetic relationships among the two closely related species, *Parascorpaenamcadamsi* and *P.moultoni*, with *Caracanthusmaculatus* (Gray, 1831) serving as the outgroup, were elucidated using MEGA 11 software (Tamura et al. 2021). Analysis applied the Kimura 2-parameter model (Kimura 1980) with 1000 bootstrap replications (Felsenstein 1985) to construct a maximum likelihood tree. All cytochrome oxidase subunit I (COI) sequences used in this study were obtained from BOLD (Barcode of Life Data System) and GenBank databases, including some generated from our previous study (Cabebe-Barnuevo et al. 2024), with accession and voucher numbers listed in Table 1.

**Table 1.** List of COI sequences utilized in the study.

Species identification	BOLD/GenBank accession number	Specimen voucher code
*Caracanthusmaculatus*	PP683413	KAUM–I. 169070
*Parascorpaenamcadamsi*	LC745944	KAUM–I. 115044
Parascorpaenamcadamsi*	PHILA1448-15	USNM 431877
Parascorpaenamcadamsi*	PHILV495-15	USNM 436361
*Parascorpaenamoultoni*	LC745946	KAUM–I. 72114

* Re-identified as *P.moultoni* based on morphological and molecular data (see Discussion).

### ﻿Comparative material

*Caracanthusmaculatus*: KAUM–I. 169070, 22.8 mm SL, Nazumado, Okago, Hachijo-jima Island, Izu Islands, Tokyo, Japan, 33°08′25″N, 139°44′11″E, 5–20 m, Y. Dewa and M. C. Sato, 10 June 2022. *Parascorpaenaarmata* (Sauvage, 1873): KAUM–I. 66443, 82.1 mm SL, off Maigo Fishing Port, Tanegashima Island, Osumi Islands, Kagoshima, Japan, 30°37′24″N, 130°56′31″E 2–8 m. *Parascorpaenaaurita* (Rüppell, 1838): KAUM–I. 161814, 44.2 mm SL, off Bandokorobana National Park, Beppu, Ei, Minami-kyushu, Kagoshima, Japan, 31°14′50″N, 130°26′E, 0.3 m, N. Kukita, 23 Oct. 2021; MZB 5564, 79.3 mm SL, off Baluran, Situbondo Regency, East Java, Indonesia, July 1984. *Parascorpaenamaculipinnis* Smith, 1957: SAIAB 395, paratype of *Parascorpaenamaculipinnis*, 43.5 mm SL, Mozambique, Sept. 1953. *Parascorpaenamcadamsi*: KAUM–I. 115044, 43.7 mm SL, south of Kiriishi Port, Suwanee-jima Island, Tokara Islands, Ryukyu Islands, Japan, 29°36′34″N, 129°42′50″E, 15–18 m, S. Tashiro et al., 26 Apr. 2018; MZB 26976, 37.1 mm SL, Batu Lompa Island, Tulehu, Ambon Island, Maluku, Indonesia, 03°35′41.2″S, 128°21′16.1″E, 15 m, K. Wibowo, 7 Nov. 2023; MZB 26978, 27.6 mm SL, Hukurila, Ambon Island, Maluku, Indonesia, 03°44′36.4″S, 128°14′29.0″E, 10 m, 12 Nov. 2023, K. Wibowo, 12 Nov. 2023; USNM 98904, holotype of *Scorpaenamcadamsi*, 37.6 mm SL, vicinity of Jola Island, Sulu Archipelago, Philippines, 06°03′45″N, 120°57′E, 36.6 m, RV *Albatross*, 5 Mar. 1908. *Parascorpaenamossambica* (Peters, 1855): ANSP 162881, 2 specimens, 25.2–38.4 mm SL, off north end of West Island, Cocos (Keeling) Islands, 12°08′36″S, 96°48′55″E, 7–8 m, W. F. Smith-Vaniz et al., 24 Feb. 1974. *Parascorpaenapicta* (Cuvier, 1829): MZB 26990, 82.4 mm SL, Nusi Island, Biak Islands, West Papua, Indonesia, 8 Aug. 1961; NSMT-P 100290, 36.0 mm SL, Dadonghai, Hainan, China, K. Matsuura. *Parascorpaenaposeidon* Chou & Liao, 2022: ANSP 51367, 95.2 mm SL, Vigan, Ilocos Sur, Luzon, Philippines, 17°34′13″N, 120°23′01″E, J. Clemens, 1923.

### ﻿Museum acronyms

**AMS**Australian Museum, Sydney

**ANSP** Academy of Natural Sciences of Drexel University, Philadelphia

**BPBM**Bernice P. Bishop Museum, Honolulu

**CAS/SU**California Academy of Sciences, San Francisco

**KAUM**Kagoshima University Museum, Kagoshima

**MZB**Museum Zoologicum Bogoriense, Cibinong

**NSMT** National Museum of Nature and Science, Tsukuba

**ROM**Royal Ontario Museum, Toronto

**SAIAB**South African Institute for Aquatic Biodiversity, Makhanda

**URM**Department of Marine Sciences, Faculty of Science, University of the Ryukyus, Okinawa

**USNM**Museum Support Center, Smithsonian Institute, National Museum of Natural History, Suitland

## ﻿Results

### ﻿ Parascorpaena moultoni

Taxon classificationAnimaliaScorpaeniformesScorpaenidae

(Whitley, 1961)

21E396E0-BE35-588D-AF92-E173E356EC1E

**Figure 1.**
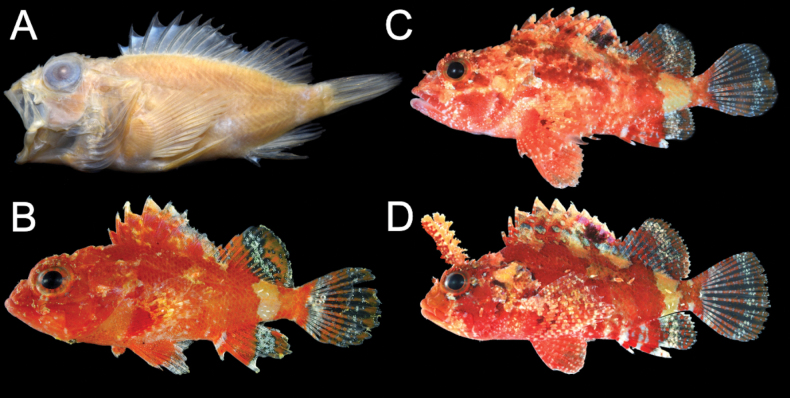
*Parascorpaenamoultoni***A**AMS IB. 5062 (holotype of *Scorpaenamoultoni*), female, 38.1 mm SL, Australia **B**KAUM–I. 82275, female, 16.7 mm SL, Japan **C**MZB 26975, male, 35.5 mm SL, Indonesia **D**KAUM–I. 124499, male, 37.0 mm SL, Japan.

**Figure 2.**
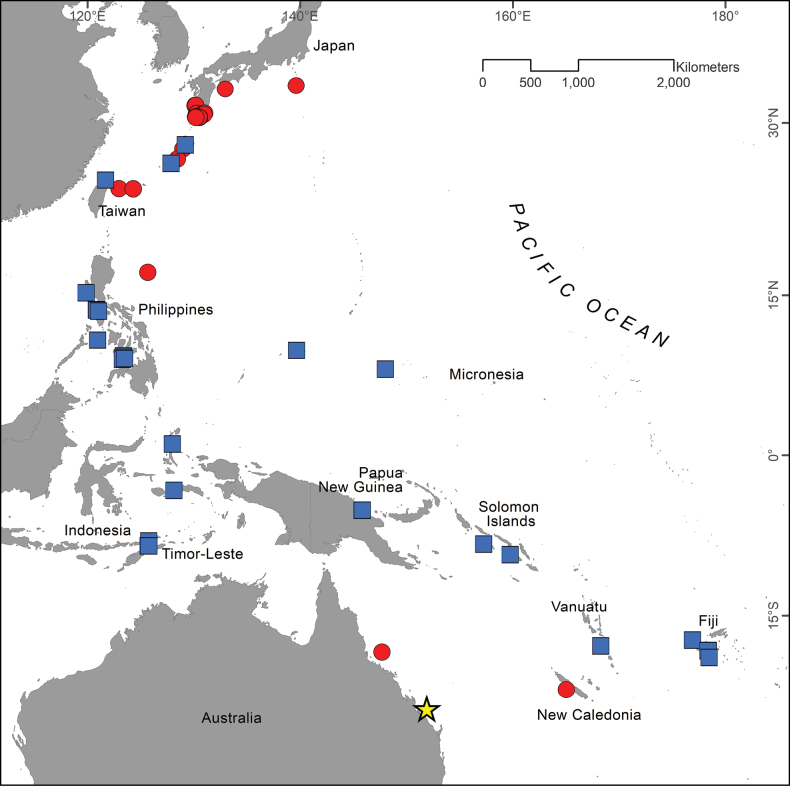
Geographic distribution of *Parascorpaenamoultoni* in the Pacific Ocean. Type locality (star); previous reports (circles); new records (squares; this study).

**Figure 3.**
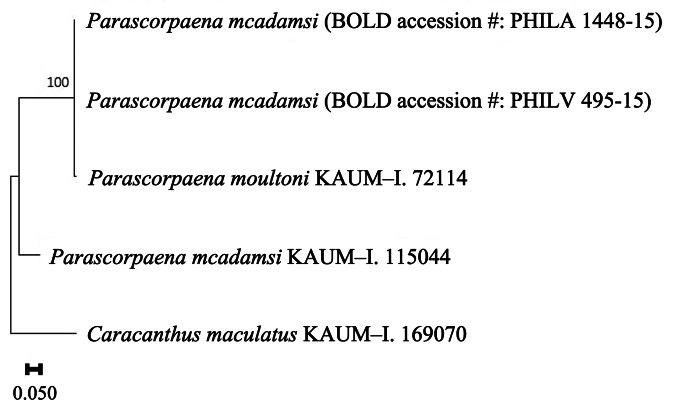
Maximum likelihood phylogenetic tree of COI sequences using the Kimura 2-parameter model, with *Caracanthusmaculatus* as outgroup. Node values indicate bootstrap support based on 1,000 replicates.

Figs 1 , 2 , 3 ; Tables 1 , 2

Scorpaena moultoni Whitley, 1961: 9, fig. 1 (type locality: north of Wilson Island, Capricorn Group, Queensland, Australia).

#### Material examined.

***Holotype*. **AMS IB. 5062 (Fig. 1A), female, 38.1 mm SL, north of Wilson Island, Capricorn Group, Queensland, Australia, 23°18′S, 151°54′E, 15 m, J. Moulton, 19 Oct. 1960. ***Paratype*.**USNM 99013, female, 27.5 mm SL, Davao Gulf, Mindanao, Philippines, 17°05′42″N, 125°39′42″E, 38 m, RV *Albatross*, 18 Mar. 1908.

#### Non-type specimens.

46 specimens, 20.4–57.1 mm SL. **Japan**: KAUM–I. 72114, male, 60.9 mm SL, north of Sanekumisaki, Setouchi, Amami-oshima Island, Amami Islands, Ryukyu Islands, 28°11′43″N, 129°11′32″E, 10–24 m; KAUM–I. 82275, female, 16.7 mm SL, San, Tokunoshima, Tokuno-shima Island, Amami Islands, 27°51′22″N, 128°58′02″E, 1–18 m, H. Motomura et al., 25 Nov. 2015; KAUM–I. 124499, male, 37.0 mm SL, off Segaura, Kushi, Bounotsu, Minami-satsuma, Kagoshima, 31°15′12″N, 130°13′35″E, 6 m, S. Morishita, 17 Dec. 2018; URM-P 4243, female, 21.1 mm SL, southwest reef off Sesoko Island, Okinawa Islands, Ryukyu Islands, 25 Oct. 1974. **Taiwan**: BPBM 23054, 43.5 mm SL, Yeh-liu, 17 m, J. E. Randall, 26 June 1978; BPBM 23385, 44.4 mm SL, southern end of Mao Pi Tou, 15 m, J. E. Randall et al., 18 July 1978. **Philippines**: BPBM 22133, male, 39.2 mm SL, Sumilon Island, Cebu, 24–26 m, J. E. Randall, 26 Aug. 1977; BPBM 22456, male, 27.8 mm SL, southwest side of Caban, Luzon, 30 m, J. E. Randall et al., 28 July 1978; BPBM 26511, female, 57.1 mm SL, Negros, south of Dumaguete, 22 m, J. E. Randall and G. W. Tribble, 9 Aug. 1978; USNM 372639, male, 26.9 mm SL, Sombrero Island, Batangas, 13°42′N, 120°49′12″E, 6 m, C. J. Ferraris, 24 Apr. 1980; USNM 372667, 37.7 mm SL, Apo Island, Negros Oriental, 09°04′38″N, 123°16′44″E, 0–40 m, V. Springer et al., 7 June 1978; USNM 372668, 2, 30.6–44.6 mm SL, Bararin Island, Palawan, 10°52′42″N, 120°56′46″E, 0–17 m, V. Springer et al., 24 May 1978; USNM 372704, 24.2 mm SL, Siquijor Island, Visayas, 09°08′30″N, 123°29′20″E, 0–6 m, V. Springer et al., 9 May 1978; USNM 431877, male, 20.4 mm SL, Maricaban Island, Batangas, 13°40′12″N, 120°51′04″E, 3–6 m, K. Carpenter et al., 29 Apr. 2014; USNM 436361, 38.2 mm SL, Verde Island, Batangas, 13°34′02″N, 121°02′31″E, 15–20 m, J. T. Williams et al., 23 Apr. 2015. **Micronesia**: BPBM 24669, 5, 20.7–31.8 mm SL, Condor Reef, Caroline Islands, 20–25 fa (36.6–45.6 m), R/V *Townsend Cromwell*, cruise 57, 23 Mar. 1972; CAS 66742, 31.8 mm SL, Ulithi Atoll, Fassarao Island, 09°55′05″N, 139°40′E, 7–25 ft (2.1–7.6 m), B. Daniel, 23 Sept. 1956. **Indonesia**: MZB 26975, male, 35.5 mm SL, Liran Island, Wetar Islands, Maluku, 08°03′03″S, 125°44′52″E, 12 m, K. Wibowo, 1 Mar. 2022; USNM 218670, 3, 23.9–26.9 mm SL, Kasa Island, Maluku, 03°18′S, 128°07′48″E, 0–1 m, V. Springer et al., 4 Mar. 1974; USNM 218671, 2, 25.4–32.1 mm SL, same data as USNM 218670; USNM 372620, 32.0 mm SL, Halmahera, North Maluku, 01°04′12″N, 127°58′48″E, H. Singou, Apr.–May 1978. **Timor-Leste**: AMS I. 46120-045, female, 34.2 mm SL, east of Dili, halfway between Hera and Metinaro, 08°31′01″S, 125°42′05″E, M. McGrouther, 23 Sept. 2012; AMS I. 46119-039, 2, female, 27.1–30.7 mm SL, off Metinaro, 08°30′25″S, 125°45′59″E, M. McGrouther, 22 Sept. 2012. **Papua New Guinea**: CAS 207711, 25.6 mm SL, Madang Harbor, Madang Province, 05°10′51″S, 145°49′41″E, 30–35 ft (9.1–10.7 m), B. Collette, 26 May 1970; USNM 380287, 40.1 mm SL, Massas Island, 05°10′18″S, 145°51′25″E, 0–18 m, V. Springer et al., 6 Nov. 1978. **Solomon Islands**: ROM 42275, male, 37.1 mm SL, Guadalcanal Island, 09°20′S, 159°45′E, P. Nichols and D. Evans, 24 Apr. 1983; USNM 266477, 32.1 mm SL, New Georgia, W. M. Chapman, 4 June 1944. **Vanuatu**: AMS I. 17472-048, female, 29.9 mm SL, Efate Island, Malapoa Peninsula, 17°44′S, 168°17′E, G. R. Allen, 22 June 1973. **Fiji**: BPBM 11354, female, 47.2 mm SL, Viti Levu Island, 18°08′28″S, 178°22′52″E, 8–20 ft (2.4–6.1 m), J. E. Randall et al., 7 Aug. 1971; BPBM 39774, male, 48.0 mm SL, Viti Levu Island, 18°09′35″S, 178°23′58″E, 35–47 ft (10.7–14.3 m), R. L. Pyle et al., 29 Jan. 2002; BPBM 39891, female, 27.7 mm SL, Viti Levu Island, 18°09′51″S, 178°24′01″E, 60 ft (18.3 m), J. L. Earle and D. F. Pence, 31 Jan. 2002; CAS 206979, female, 41.1 mm SL, Viwa Island, 17°12′S, 176°54′E, 70–100 ft (21.3–30.5 m), V. Springer et al., 27 May 1982; CAS 214143, male, 52.5 mm SL, female, 42.8 mm SL, Viti Levu Island, 18°08′38″S, 178°22′49″E, 63 ft (19.2 m), D. W. Greenfield and K. Cole, 2 June 1999; CAS 218624, male, 39.3 mm SL, female, 24.5 mm SL, Viti Levu Island, 18°09′02″S, 178°21′37″E, 10–26 ft (3.0–7.9 m), D. W. Greenfield et al., 12 Feb. 2002; ROM 51930, female, 35.5 mm SL, Great Astrolabe Reef, 18°46′S, 178°28′05″E, R. Winterbottom et al., 5 Apr. 1983.

#### Diagnosis.

A species of *Parascorpaena* with the following characters: pectoral-fin rays 15–17 (usually 15 or 16); pored lateral-line scales 20–22 (mode 21); scale rows in longitudinal series 40–48 (41); scale rows above lateral line 5–8 (7); scale rows below lateral line 10–12 (12); scale rows between sixth dorsal-fin spine base and lateral line 5 or 6 (6); scale rows between last dorsal-fin spine base and lateral line 5 or 6 (5); total gill rakers 12–14 (12); two suborbital ridges, each with one spine; origin of first suborbital ridge posterior to origin of second ridge; interorbital ridges well developed posteriorly from middle of eye, forming a broad loop on rear end, enclosed concavity relatively shallow; occipital pit not developed, almost flat; no scales along dorsal- and anal-fin soft ray bases; black botch on spinous portion of dorsal fin in males, usually found along 7^th^–10^th^ spines.

#### Description.

Measurements of examined specimens, expressed as percentages of SL and HL, provided in Table 2. Body size small, compressed, with numerous widely scattered papillae; body depth greatest at pelvic-fin spine base. Lateral line complete, pored lateral-line scales continuing onto caudal-fin base (except in small specimens). Head size moderate, length less than half SL, with scattered papillae. Snout moderately blunt; dorsal profile steep. Eyes large, diameter slightly greater than snout length. Mouth large, maxilla extending just below posterior margin of eye. Four prominent pairs of mandibular pores on dentary, first pore positioned just behind tip of lower jaw, second along anterior lacrimal spine, third along posterior lacrimal spine, and fourth behind posterior lacrimal spine (before posterior end of maxilla). Teeth on upper jaw villiform; teeth on lower jaw varying among specimens, from entirely villiform to villiform with enlarged or canine-like teeth on frontal area; teeth on palatines small, villiform, in bands; teeth on vomerine villiform, in V-shaped patch. Body entirely covered with cycloid scales (rarely with weak ctenii); body scales not extending to dorsal- and anal-fin soft ray membranes; head mostly naked, a few, small scales behind posterior margin of eye, along posterior end of operculum, and just behind opercular spines; dentary smooth, naked.

**Table 2.** Counts and proportional measurements (expressed as percentages of standard and head lengths) of *Parascorpaenamoultoni*.

	AMS IB 5062	USNM 99013	Non-type specimens (*n* = 23)
	Holotype of *S.moultoni*	Paratype of *S.mcadamsi*	
Standard length (SL)	38.1	27.5	20.7–57.1
Head length (HL)	17.4	11.4	9.8–25.2
**Counts**			
Dorsal-fin rays	XII, 9	XII, 9	XII, 9
Anal-fin rays	III, 5	III, 5	III, 5
Pectoral-fin rays (left/right sides)	15/15	16/damaged	15–17/15–17 (mode 16/16)
Pored lateral-line scales	21	20	20–22 (21)
Scale rows in longitudinal series	43	42	40–48 (43)
Scale rows above lateral line	7	6	5–8 (6)
Scale rows below lateral line	10	10	10–12 (11)
Scale rows between 6^th^ dorsal-fin spine base and lateral line	5	5	5–6 (6)
Scale rows between last dorsal-fin spine base and lateral line	5	5	5–6 (5)
Pre-dorsal-fin scale rows	3	4	3–4 (3)
Total gill rakers	11	13	11–14 (12)
Gill rakers (lower + hypobranchial)	7	8	7–10 (8)
Gill rakers (upper)	4	5	4–5 (4)
**Measurements (% SL)**			
Body depth at pelvic-fin spine base	36.2	36.5	32.4–38.2
Body depth at first anal-fin spine base	30.2	28.3	26.2–33.7
Body width	15.8	13.7	8.2–20.1
Predorsal-fin length	39.4	38.0	36.5–49.3
Preanal-fin length	73.4	73.8	66.3–76.1
Prepelvic-fin length	52.2	41.3	35.2–49.3
Pectoral-fin ray length	32.8	damaged	28.7–45.0
1^st^ dorsal-fin spine length	4.8	8.5	3.5–9.7
2^nd^ dorsal-fin spine length	8.8	13.0	9.2–13.9
3^rd^ dorsal-fin spine length	14.8	17.3	14.2–20.3
4^th^ dorsal-fin spine length	17.6	20.1	15.1–21.1
5^th^ dorsal-fin spine length	17.8	19.4	15.6–20.9
11^th^ dorsal-fin spine length	9.7	10.3	7.6–12.7
12^th^ dorsal-fin spine	14.4	16.3	12.1–17.5
Longest dorsal-fin soft ray length	18.4	damaged	16.9–22.4
1^st^ anal-fin spine length	7.0	10.5	8.5–12.3
2^nd^ anal-fin spine length	21.2	23.0	16.9–27.1
3^rd^ anal-fin spine length	16.5	18.7	15.5–21.2
Longest anal-fin soft ray length	20.2	21.0	18.7–24.9
Pelvic-fin spine length	16.5	17.4	15.7–19.2
Longest pelvic-fin soft ray length	25.2	26.1	22.1–30.2
Caudal-fin length	23.1	24.1	24.8–32.1
Caudal-peduncle depth	9.5	9.8	7.5–11.5
Caudal-peduncle length	13.4	18.2	9.6–16.7
Head length	45.6	41.4	42.2–51.6
**Measurements (% HL)**			
Head width	49.4	55.8	31.4–50.0
Snout length	35.6	27.2	23.4–33.5
Eye diameter	33.2	34.5	28.7–38.8
Interorbital width at vertical midline of eye	12.4	15.4	10.9–17.2
Interorbital width at posterior end of preocular spine base	9.8	14.8	9.7–14.7
Upper-jaw length	46.7	53.3	44.2–55.2
Maxillary depth	14.0	15.6	14.8–20.5
Postorbital length	42.2	46.7	45.7–53.8
Distance between opercular spine tips	13.4	14.5	11.1–15.3
Supraocular-tentacle length	6.9	absent	3.2–30.6

Dorsal-fin spines usually 12 (rarely 13) connected to soft rays; fourth or fifth spine longest; sixth to 11^th^ spine gradually decreasing in size; 12^th^ spine elongated, followed by nine (rarely 8) branched soft rays; second dorsal-fin soft ray usually longest; 7^th^–10^th^ dorsal spines bearing black blotch in male specimens. Anal-fin spines three, second spine longest; five branched soft rays. Bases of both dorsal- and anal-fin soft rays without scales. Pectoral-fin rays usually 15 or 16 (rarely 17); first (uppermost) ray simple, unbranched; second to fifth or sixth ray branched; lower rays thickened, unbranched; tips of fins reaching beyond origin of anal-fin spine. All pectoral-fin rays in small specimens (e.g., <20.0 mm SL) unbranched. Pelvic-fin length variable, not or just reaching anal-fin spine base.

Nasal spines positioned bilaterally on nasal ridge, extending slightly beyond rim. Preocular spine relatively thick, with broad base, located anteriorly within orbital region. Interorbital ridge originating either from above anterior half of eye, or along preocular spine base, extending beyond posterior eye margin; rear end of ridge forming a broad, very shallow interorbital loop. Occipital pit indistinct, almost flat. Supraocular and postocular spines situated above orbital region, close to one another. Supraocular spines each occasionally bearing a single, variably sized tentacle, sometimes small but usually longer than eye diameter. Tympanic spines simple, just behind postocular spine, usually separated by distance greater than that between parietal and nuchal spines. Parietal and nuchal spines simple, close to one another; parietal spine originating posterior to origin of pterotic spine; nuchal spine originating just behind parietal spine. Sphenotic spine just behind posterior margin of eye, small, usually as unevenly sized pair (rarely single). Pterotic spine simple, attached to skin, situated just behind sphenotic spine. Upper and lower post-temporal spines well-developed, upper spine shorter than and positioned just above lower spine, lower spine situated between pterotic and supracleithral spines. Suborbital with two distinct ridges, a suborbital spine on each ridge. Lacrimal bone dorsally with two distinct ridges; anterior lacrimal ridge located before anterior eye margin, longer than posterior lacrimal ridge; posterior ridge located just behind origin of first suborbital ridge (just below ventral eye margin). Anterior and posterior lacrimal spines located along ventral region of lacrimal bone, simple with no additional spinous points; anterior lacrimal spine prominently antrorse, tip not extending beyond lower lip; posterior lacrimal spine distinctly anteriorly oriented in larger specimens (>24.5 mm SL), ventrally oriented with forward curvature in smaller specimens to ~ 20 mm SL, postero-ventrally oriented in specimens <20 mm SL. Preopercular spines five; first and second along posterior margin of maxilla, covered with thick skin, usually with small tentacles; third to fifth exposed, progressively longer, fifth longest with small anterior supplemental preopercular spine. Opercular spines just behind pre-opercular margin; upper opercular spine slightly longer than lower opercular spine. Supracleithral spine single, short, located between upper and lower post-temporal spines (closer to latter). Cleithral spine simple, base covered by operculum; spinous point not extending beyond posteriormost tip of operculum. Postorbital spine usually absent; if present, lump-like, lacking a spinous point. Median interorbital ridge, coronal spine, ridge on lateral surface of maxilla, antero-dorsal lacrimal spines, and lateral lacrimal spine all absent.

#### Color of fresh specimens.

Based on color photographs of three specimens deposited at KAUM and MZB (Fig. 1B–D). Body variegated, predominantly orange-red with interspersed darker red and white blotches. White areas particularly apparent near dorsal region and caudal peduncle. Head predominantly reddish, with numerous orange and white blotches; underside white with reddish mottling along jawline. Spinous portion of dorsal fin variegated orange, reddish, and white; a distinct black blotch on 7^th^–10^th^ spines in males. Soft-rayed portion of dorsal fin translucent white, with poorly defined orange-red and white blotches. Pectoral fin translucent with poorly defined reddish and white blotches. Pelvic fin orange-red at base, transitioning to white distally. Anal fin primarily orange-red, with poorly defined white blotches. Caudal fin translucent with three orange-red bands; first band on base, second in middle, and third on distal margin.

#### Color of preserved specimens.

Body and ventral surface yellowish-white. Head typically white; some specimens with poorly defined dark blotches. Spinous portion of dorsal fin translucent; distinct black blotch retained in males. Soft-rayed portion of dorsal fin usually translucent; some specimens with poorly defined dark blotches. Anal, pectoral, and pelvic fins usually entirely translucent, with poorly defined dark blotches in some specimens. Caudal fin generally translucent, dark bands apparent in some specimens.

#### Distribution.

*Parascorpaenamoultoni* has been previously recorded from Queensland, Australia (Whitley 1961; Allen and Cross 1989), New Caledonia (Motomura et al. 2011; Fricke et al. 2011, 2015), and Japan (Motomura 2013, 2019, 2022, 2023; Motomura and Harazaki 2017; Kimura et al. 2017; Nakae et al. 2018; Mochida and Motomura 2018; Tanaka et al. 2020; Jeong and Motomura 2021; Tsuno et al. 2022; Mochizuki et al. 2023). The voucher specimens examined here extended the confirmed distribution of the species to include Taiwan, the Philippines, Micronesia, Indonesia, Timor-Leste, Papua New Guinea, the Solomon Islands, Vanuatu, and Fiji (Fig. 2).

## ﻿Discussion

This study largely corroborated Whitley’s (1961) original description of *Scorpaenamoultoni*. However, although Whitley (1961) described the holotype of *Scorpaenamoultoni* as lacking supraorbital tentacles (supraocular tentacles in this study), examination of the holotype revealed the presence of short tentacles. Furthermore, the condition of the supraocular tentacles varied among other specimens, sometimes being small but more commonly elongated, usually exceeding the eye diameter. Dental morphology also varied among specimens, the upper-jaw teeth being consistently villiform, whereas those on the lower jaw varied from entirely villiform to enlarged or canine-like teeth anteriorly. The sexual dimorphism seen in *P.mcadamsi*, male specimens having canine-like teeth anteriorly and females only villiform teeth, was not apparent in *P.moultoni* (see Mochizuki et al. 2023). Although Whitley (1961) described undeveloped sphenotic spines, the holotype of *P.moultoni* in fact had two well-developed sphenotic spines. Moreover, the non-type specimens examined typically exhibited one or two sphenotic spines, being unequally-sized in the case of two present. Whitley (1961) also noted that the “upper profile behind the eye is not deeply notched”, which refers to the occipital pit, described here as indistinct.

Whitley (1961) initially differentiated *P.moultoni* from its congeners based on several features, including pale coloration, naked head, short maxillary, large scales, absence of dermal flaps, and presence of a few predorsal scales. However, these characters also occur in other species of *Parascorpaena*, making them unsuitable for reliable identification of the former. Although Whitley (1961) also mentioned that *P.moultoni* had two suborbital spines, he did not regard that feature as a significant distinguishing trait. However, after *P.moultoni* had been synonymized with *P.mcadamsi* by Allen et al. (2006), it was later reinstated as a distinct species by Motomura et al. (2011), who emphasized the number of suborbital spines as the key differentiating character from *P.mcadamsi*.

Among the eight valid species of *Parascorpaena* (see Cabebe-Barnuevo et al. 2024), only *P.mcadamsi* and *P.moultoni* exhibit sexual dichromatism, characterized by a black blotch on the 7^th^–10^th^ dorsal-fin spines in males, such being absent in females (Eschmeyer 1986; Poss 1999; Poss and Motomura 2022; Mochizuki et al. 2023). Examination of the type specimens of *P.mcadamsi* revealed that one of the two paratypes (USNM 99013) was, in fact, *P.moultoni*, distinguishable from the holotype by the number of suborbital spines – two in USNM 99013 and three in the holotype (USNM 98904).

*Parascorpaenamoultoni* is clearly differentiated from *P.armata*, *P.mossambica*, and *P.poseidon* by its two suborbital spines and indistinct occipital pit, whereas the latter three species possess three suborbital spines and a well-developed occipital pit. Although *P.moultoni* shares the same number of suborbital spines with *P.aurita* and *P.picta*, it differs in having a single spine on each suborbital ridge, compared to the absence of a spinous point on the first ridge and presence of two spines on the second ridge in the latter two species, and can further be differentiated by the absence of scales below the dorsal- and anal-fin soft ray bases, which are present in *P.aurita* and *P.picta*. Moreover, while *P.moultoni* resembles *P.maculipinnis* and *P.mcadamsi* in having an undefined occipital pit, the interorbital ridge posteriorly forming a loop, naked dorsal- and anal-fin soft ray bases, and the origin of the first ridge posterior to the second, the former has only two suborbital spines, compared to the three spines (one on the first ridge and two on the second) found in the latter two species. Consequently, this study affirms that *P.moultoni* can be consistently distinguished from other species of *Parascorpaena* based on occipital pit morphology and the number of suborbital spines.

Cabebe-Barnuevo et al. (2024) compared the orientation of the posterior lacrimal spine (PLS) across differing size categories within the genus *Parascorpaena* and identified three distinct orientations. In the largest individuals, with standard lengths (SLs) of 118.0 mm and 121.4 mm, the PLS was oriented anteriorly. However, in smaller individuals with SLs to 21 mm, the PLS was oriented ventrally with an anterior curvature. The smallest individuals, measuring less than 20 mm SL, exhibited postero-ventral orientation of the PLS. Similar differing orientations of the PLS with size categories were found in *P.moultoni*, although the largest recorded specimen of that species was substantially smaller at 57.1 mm SL (this study). Specimens of 24.5–57.1 mm SL had an anteriorly oriented PLS, smaller specimens being characterized by either ventral (e.g., 21.08 mm SL) or postero-ventral (e.g., <20 mm SL) orientation. Clearly, similar growth-related variations in PLS orientation are characteristic of species of *Parascorpaena*, regardless of the maximum size attainable by each.

Cytochrome oxidase subunit I (COI) sequences from KAUM specimens, deposited in GenBank, were utilized to verify the identification of some sequences of *Parascorpaena*. This analysis (Fig. 3) specifically focused on sequences from BOLD and GenBank data originally identified as *P.mcadamsi*, but which were subsequently determined to represent *P.moultoni*. Given that *P.moultoni* was previously regarded as a synonym of *P.mcadamsi*, it is unsurprising that some sequences in public databases (e.g., BOLD) initially identified as *P.mcadamsi* are actually of *P.moultoni*. This study confirmed that sequences with accession numbers PHILA 1448-15 and PHILV 495-15, initially identified as *P.mcadamsi*, represent a single clade together with the sequence of a verified specimen of *P.moultoni* (KAUM–I. 72114), being distinct from the sequence of a verified specimen of *P.mcadamsi* (KAUM–I. 115044). Morphological examination of the voucher specimens for both PHILA 1448-15 and PHILV 495-15 revealed that both specimens had only two suborbital spines, an interorbital ridge forming a loop, an indistinct occipital pit, a black blotch on the 7^th^–10^th^ dorsal-fin spines, a strongly anteriorly oriented PLS, and cycloid scales, all being consistent with *P.moultoni*. Clearly, gene sequences in public databases, together with associated voucher specimens, should remain under review so as to lessen the likelihood of mistakenly identified sequences and problems arising therefrom.

## Supplementary Material

XML Treatment for Parascorpaena moultoni

## ﻿Additional information

### Author ORCIDs

Roxanne Cabebe-Barnuevo https://orcid.org/0000-0001-6518-7986

Kunto Wibowo https://orcid.org/0000-0003-4465-3022

Hiroyuki Motomura https://orcid.org/0000-0002-7448-2482

### Data availability

All of the data that support the findings of this study are available in the main text.
